# Intestinal epithelial tuft cell induction is negated by a murine helminth and its secreted products

**DOI:** 10.1084/jem.20211140

**Published:** 2021-11-15

**Authors:** Claire Drurey, Håvard T. Lindholm, Gillian Coakley, Marta Campillo Poveda, Stephan Löser, Rory Doolan, François Gerbe, Philippe Jay, Nicola Harris, Menno J. Oudhoff, Rick M. Maizels

**Affiliations:** 1 Wellcome Centre for Integrative Parasitology, Institute of Infection, Immunity and Inflammation, University of Glasgow, UK; 2 Centre of Molecular Inflammation Research, Department of Clinical and Molecular Medicine, Norwegian University of Science and Technology, Trondheim, Norway; 3 Department of Immunology and Pathology, Central Clinical School, Monash University, Melbourne, Australia; 4 Institut de Génomique Fonctionnelle, University of Montpellier, Centre national de la recherche scientifique UMR-5203, Institut National de la Santé et de la Recherche Médicale U1191, Montpellier, France

## Abstract

Helminth parasites are adept manipulators of the immune system, using multiple strategies to evade the host type 2 response. In the intestinal niche, the epithelium is crucial for initiating type 2 immunity via tuft cells, which together with goblet cells expand dramatically in response to the type 2 cytokines IL-4 and IL-13. However, it is not known whether helminths modulate these epithelial cell populations. In vitro, using small intestinal organoids, we found that excretory/secretory products (*Hp*ES) from *Heligmosomoides polygyrus* blocked the effects of IL-4/13, inhibiting tuft and goblet cell gene expression and expansion, and inducing spheroid growth characteristic of fetal epithelium and homeostatic repair. Similar outcomes were seen in organoids exposed to parasite larvae. In vivo*, H. polygyrus* infection inhibited tuft cell responses to heterologous *Nippostrongylus brasiliensis* infection or succinate, and *Hp*ES also reduced succinate-stimulated tuft cell expansion. Our results demonstrate that helminth parasites reshape their intestinal environment in a novel strategy for undermining the host protective response.

## Teaser

The intestinal helminth *Heligmosomoides polygyrus* suppresses development of epithelial tuft cells, which are essential for the type 2 immune response, through secreted factors that block tuft cells both in intestinal organoids and when administered in vivo.

## Introduction

Helminth infections cause major neglected tropical diseases, afflicting >20% of the world’s population with soil-transmitted helminths alone (Jourdan et al., 2018; Pullan et al., 2014). Unlike many pathogens, parasitic helminths establish long-lasting infestations, frequently evoking minimal inflammatory reaction. This has been attributed to the release of multiple immunomodulatory molecules throughout infection, broadly referred to as excretory-secretory (ES) products (Lightowlers and Rickard, 1988; Maizels et al., 2018). Most studies have focused on how ES constituents affect immune cells, for instance the TGF-β mimic of *Heligmosomoides polygyrus* (TGM), which induces immunosuppressive regulatory T cells (Cook et al., 2021; Johnston et al., 2017; White et al., 2021). However, intestinal helminths localize to the intestinal lumen in close association with the epithelial layer, and the direct effects of helminths or their products on the epithelium are poorly understood.

The epithelium initially alerts the immune system to incoming parasites, producing the alarmins IL-25, IL-33, and thymic stromal lymphopoietin in response to tissue damage caused by helminths (Artis and Grencis, 2008) such as *H. polygyrus*, which invades the submucosa at the larval stage before returning to the lumen as an adult (Reynolds et al., 2012). Alarmins recruit innate immune cells and APCs to the infected area, and activate type 2 immune responses, including CD4^+^ T helper type 2 (Th2) cell effectors (Inclan-Rico and Siracusa, 2018). Parasite-secreted proteins have been identified that block epithelial alarmin signals, such as *H. polygyrus* alarmin release inhibitor (HpARI), which binds IL-33 to prevent its release from damaged cells (Osbourn et al., 2017). However, ES constituents with direct effects on epithelial cells themselves have yet to be identified, despite helminths inducing extensive physiological changes to the gut (McKay et al., 2017).

The intestinal epithelium is remodeled drastically during helminth infection, driven by the type 2 signature cytokines IL-4 and IL-13. These drive the “weep and sweep” response with increased epithelial mucus production and intestinal muscular contractility required for parasite expulsion, and expansion of epithelial goblet, Paneth, and tuft cells (Gerbe et al., 2016; Howitt et al., 2016; Kamal et al., 2002; Sharpe et al., 2018). Recently, tuft cells have been recognized as central orchestrators in this response, producing IL-25, leukotrienes, and other mediators that activate ILC2s to produce IL-13 (Billipp et al., 2021; McGinty et al., 2020; von Moltke et al., 2016). IL-13 stimulates epithelial cells in a positive feedback loop to increase tuft and goblet cell numbers. IL-4, which shares the receptor subunit IL-4Rα with IL-13, can also induce tuft cell hyperplasia (Gerbe et al., 2016).

To investigate the response of the intestinal epithelium to helminth ES products, we first exposed murine small intestinal organoids to *H. polygyrus* ES products (*Hp*ES) in combination with Th1 or Th2 cytokines, to mimic the effects of infection. RNA sequencing (RNA-seq) of these organoids revealed a down-regulation of tuft cell–associated genes by *Hp*ES, alongside inhibition of tuft cell expansion. In vivo*,* tuft cell induction by both succinate and the nonresident helminth *Nippostrongylus brasiliensis* was reduced in the presence of *H. polygyrus*. *Hp*ES also exerted generalized effects on organoid development, altering expression of key transcription factors and promoting a spheroid morphology. Together, these data demonstrate that *H. polygyrus* directly affects the development of the intestinal epithelium via ES products.

## Results and discussion

### Small intestinal organoid gene expression is differentially regulated by immune cytokines and *Hp*ES

To model the impact of helminth products on the intestinal epithelium, we first analyzed the transcriptional profile of mouse small intestinal organoid cultures exposed to ES molecules (*Hp*ES) released by the intestinal helminth *H. polygyrus.* Established organoid cultures were stimulated with IFN-γ as the main driver of type 1 responses, or a combination of IL-4 and IL-13 to induce type 2 responses, in the presence or absence of *Hp*ES. Stimulations were performed over 24 h in replicate organoids derived from four different C57BL/6 individual mice. RNA was extracted from each replicate and subjected to bulk RNA-seq analyses.

**Figure 1.**
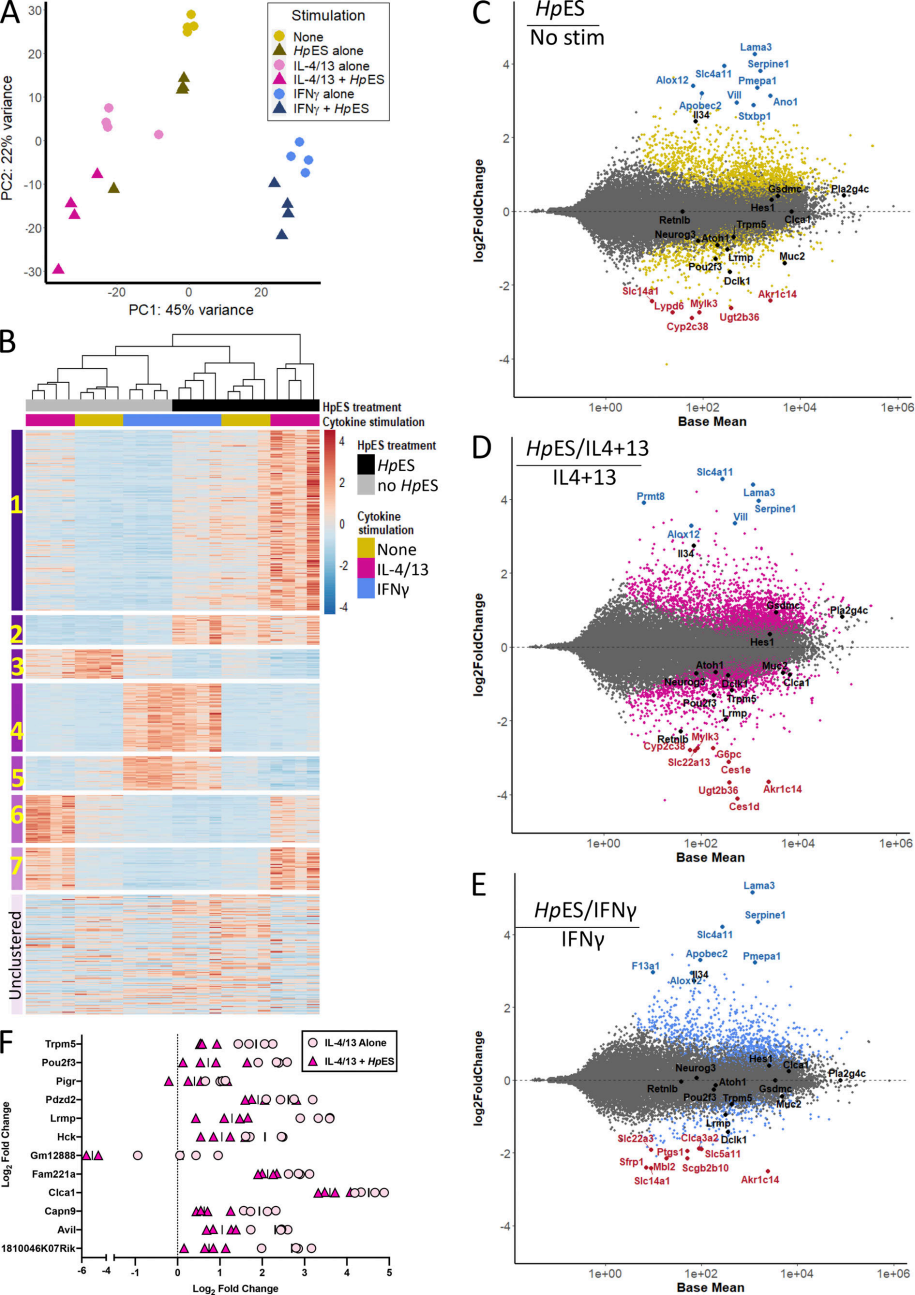
**Gene expression in *Hp*ES- and cytokine-treated small intestinal organoids.** Organoid cultures grown from duodenal crypt cells, taken from four individual C57BL/6 mice, were stimulated in the presence or absence of the type 1 cytokine IFN-γ, the type 2 cytokines IL-4 and IL-13 (IL-4/13), and/or the ES products of adult *H. polygyrus* parasites (*Hp*ES). After 24 h, RNA was extracted from each replicate, and all 24 samples were subjected to parallel RNA-seq analyses. Patterns of gene expression were then analyzed. **(A)** Principal component analysis plot showing transcriptomes of stimulated organoids projected onto two dimensions; each condition is represented by four replicates derived from four individual mice. Principal component analysis data were produced from log-transformed normalized counts in the DESeq2 package (Love et al., 2014). **(B)** Heat map of the top 1,000 DEGs, organized into seven gene clusters, and a group of unclustered genes. Z-scores of normalized count values are indicated by coloring from blue (lowest) to red (highest), based on data from four replicates per group, each individually presented. **(C–E)** MA plots showing log_2_ fold change (*M*) plotted against log of mean normalized expression counts (*A*) for the comparisons of no stimulation versus *Hp*ES alone (C), IL-4/13 alone versus IL-4/13 + *Hp*ES (D), and IFN-γ alone versus IFN-γ + *Hp*ES (E). Data from four replicates per group were pooled; genes with adjusted P value < 0.05 are colored. The top 10 DEGs by log_2_ fold change that have known function are annotated, with those increased in the presence of *Hp*ES shown in blue and those decreased in red. Additional genes of interest are shown in black. DEGs were calculated in DESeq2. **(F)** Expression levels of 12 genes from cluster 6 showing the most significant (by adjusted P value) differential expression in the presence of *Hp*ES. Graphs show normalized counts/log_2_ fold change compared with no stimulation control from four independent biological replicates, for organoid cultures exposed to IL-4/13 alone (pink circles) or IL-4/13 + *Hp*ES (magenta triangles). Stim, stimulation.

By principal component analysis, samples from the same stimulation conditions clustered together (Fig. 1 A), with the majority of variance driven by cytokine stimulation (PC1, at 45%); notably, treatment with type 1 or type 2 cytokines induced diametrically opposite effects on PC1 compared with unstimulated controls. The addition of *Hp*ES caused a consistent shift in PC2 in all groups, with the same directionality in the presence or absence of cytokines, explaining >20% of the remaining variance. These results support the hypothesis that epithelial cells are highly responsive to both type 1 and type 2 cytokines and demonstrate that these responses can be significantly affected by helminth products.

Just under 34,000 (33,857) different transcripts were identified, and 15,000 were identified as differentially expressed genes (DEGs) using a likelihood ratio test across all conditions. A cluster analysis of DEGs defined seven expression patterns, and a heatmap of the 1,000 most altered genes in the study is presented in Fig. 1 B, demonstrating that *Hp*ES has profound gene-specific effects on intestinal epithelial cells. Pairwise comparisons between the different conditions revealed that while addition of IL-4/13 or IFN-γ alone modulated 1,150 and 1,673 DEGs, respectively, organoids exposed to *Hp*ES had a far greater number of DEGs than their equivalents stimulated with cytokines alone, with >6,700 (3,867 up-regulated and 2,913 down-regulated) resulting from the addition of *Hp*ES to the type 2 cytokines. The gene-specific effects of *Hp*ES are shown in organoid cultures without added cytokines (Fig. 1 C), or added IL-4/IL-13 (Fig. 1 D) and IFN-γ (Fig. 1 E), presented as MA plots of log fold-change (M) versus mean abundance (A). The more marked effect of *Hp*ES in the type 2 setting of IL-4/13 stimulation compared with type 1/IFN-γ (Fig. 1, B and D) is consistent with adaptation by this helminth parasite to modulate the mode of immunity that would lead to its expulsion (Reynolds et al., 2012).

**Figure S1.**
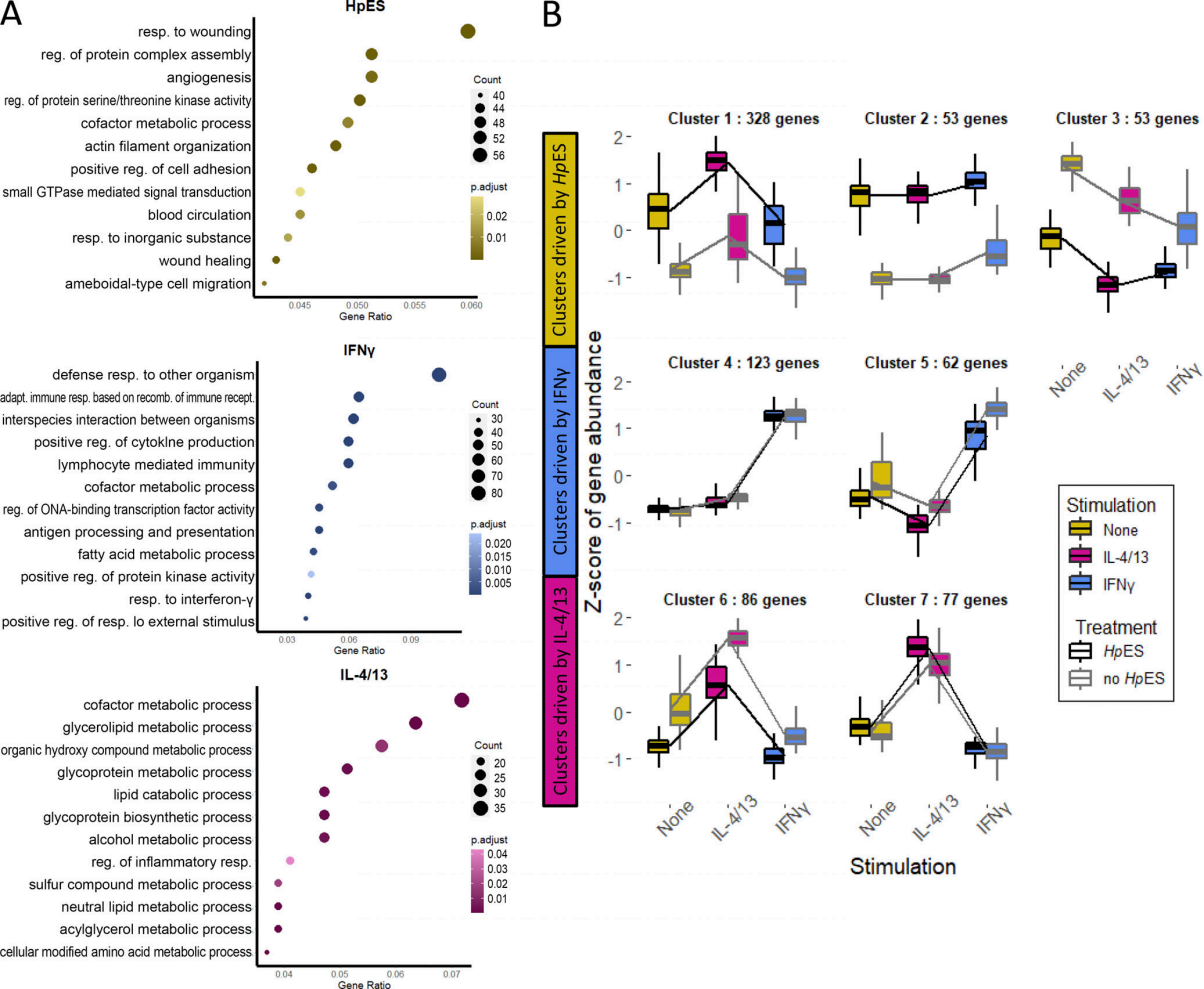
**GO terms for DEGs. (A)** *Hp*ES and cytokine treatment of small intestinal organoids. Top 12 GO terms, by gene ratio, for DEGs from *Hp*ES, IL-4/13, and IFN-γ individual treatments. Data are based on analyses of four biological replicates, composed of a total of 24 samples analyzed in parallel by RNA-seq. DEGs were selected as those with a P-adjusted value of <0.01. The enrichGO function from ClusterProfiler (Yu et al., 2012) was used to identify enriched GO terms, followed by ReViGO (Supek et al., 2011) to remove redundant GO terms. The top 12 GO terms were then selected using gene ratio (number of genes associated with GO term in list/total number of genes in list). **(B)** Responses of identified gene clusters to *Hp*ES, IL-4/13, and IFN-γ. Gene sets were split based on treatment (± *Hp*ES) and stimulation (IL-4/13, IFN-γ, or none). Clusters were identified using degPatterns, a part of the DEGreport package (Pantano et al., 2020). Adapt., adaptive; GTPase, guanosine triphosphatase; rec., reecombinant; reg., regulation; resp., response.

We next analyzed gene ontology (GO) terms, which reflected induction of inflammatory responses by IFN-γ and multiple metabolic pathways by the type 2 cytokines; notably, treatment with *Hp*ES preferentially activated wound healing and angiogenesis pathways (Fig. S1 A), a finding of interest with respect to the tissue-invasive life cycle of *H. polygyrus*. The individual genes most up-regulated by *Hp*ES treatment of organoids included *Serpine1*, *Lama3 *(Laminin subunit α 3) and *Vill *(villin-like), all involved in cell adhesion, suggesting induction of structural changes in the organoids by *Hp*ES (Fig. 1 C). IL-4/13 alone up-regulated two goblet cell products, *Clca1*, a calcium-activated chloride channel regulator, and *Retnlb*, which encodes resistin-like molecule (RELM)-β, known to impair *H. polygyrus* feeding (Herbert et al., 2009). Both genes are down-regulated by the addition of *Hp*ES (Fig. 1 D). In agreement with investigated GO terms, we observed in all samples exposed to *Hp*ES a marked increase in arachidonate 12-lipoxygenase (*Alox12*), which may convert arachidonic acid into repair-promoting lipoxins and resolvins (Esser-von Bieren, 2019), as well as phospholipase A2 (*Pla2g4c*), which is responsible for forming arachidonic acid from phospholipid membranes.

### IL-4/13–responsive genes down-regulated by *Hp*ES include markers of intestinal tuft cells

**Figure S2.**
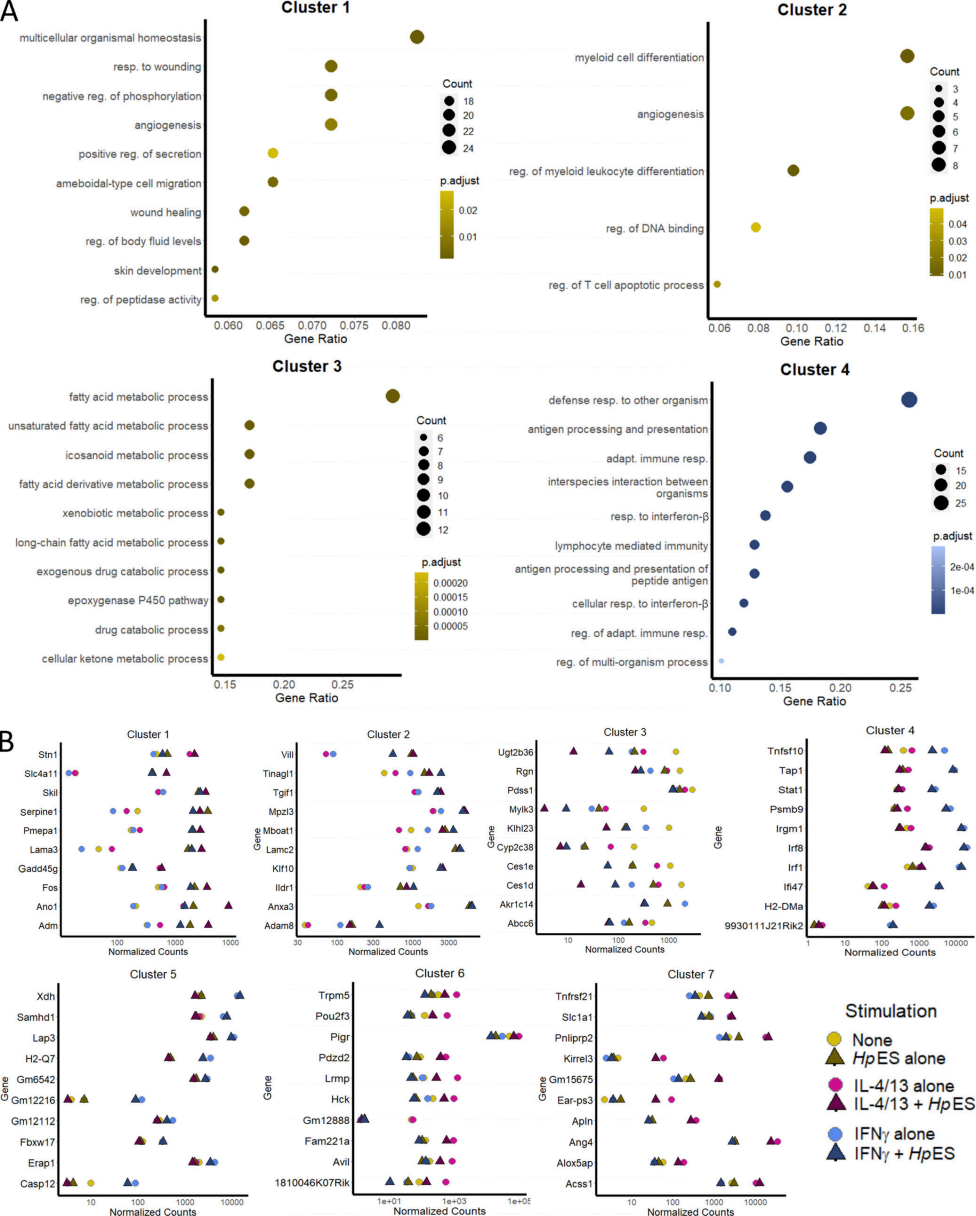
**Cluster analysis of *Hp*ES modulated gene expression. (A)** Top 5 or 10 GO terms by gene ratio for each cluster. Only clusters 1–4 were identified as having corresponding GO terms using enrichGO from ClusterProfiler, and only five GO terms were returned for cluster 2. Redundant GO terms were removed using ReViGO before plotting. **(B)** Mean normalized counts for the top 10 genes from each cluster by P-adjusted values. Adapt., adaptive; reg., regulation; resp., response.

Clustering analysis on the top 1,000 DEGs (ranked by P-adjusted value), grouping genes according to their expression profile across the different stimulation, identified seven clusters with different expression profiles containing >50 genes (Fig. S1 B). The largest, cluster 1, corresponded to 328 genes that were up-regulated by both *Hp*ES and IL-4/13, many of which were represented by GO terms for homeostatic maintenance and the response to wounding (Fig. S2 A). Cluster 2 also contained genes up-regulated by *Hp*ES, irrespective of the cytokine milieu. Other clusters of interest grouped together genes down-regulated by *Hp*ES under all conditions (cluster 3), and clusters up-regulated by IFN-γ that were unaffected (cluster 4) or down-modulated (cluster 5) by *Hp*ES. The top 10 DEGs for each cluster, and their expression levels in each stimulatory condition, are shown in Fig. S2 B.

We then focused on cluster 6, containing 86 genes up-regulated by IL-4/13 treatment but repressed by *Hp*ES, including *Lrmp*, *Pou2f3*, and *Trpm5* (Fig. 1 F), which in the intestinal epithelium are specific for tuft cells, a chemosensory cell type that differentiates in response to IL-13 (Gerbe et al., 2016; von Moltke et al., 2016). Mice lacking either *Pou2f3* (Gerbe et al., 2016) or *Trpm5* (Howitt et al., 2016) are defective in their ability to expel helminth parasites. The goblet cell marker *Clca1* (Leverkoehne and Gruber, 2002) is also present in this set of DEGs. The down-regulation of these markers suggests that *H. polygyrus* may interfere with intestinal development to inhibit cell types involved in helminth elimination.

### *Hp*ES and L3 larvae repress tuft cell expansion in small intestinal organoids

**Figure 2.**
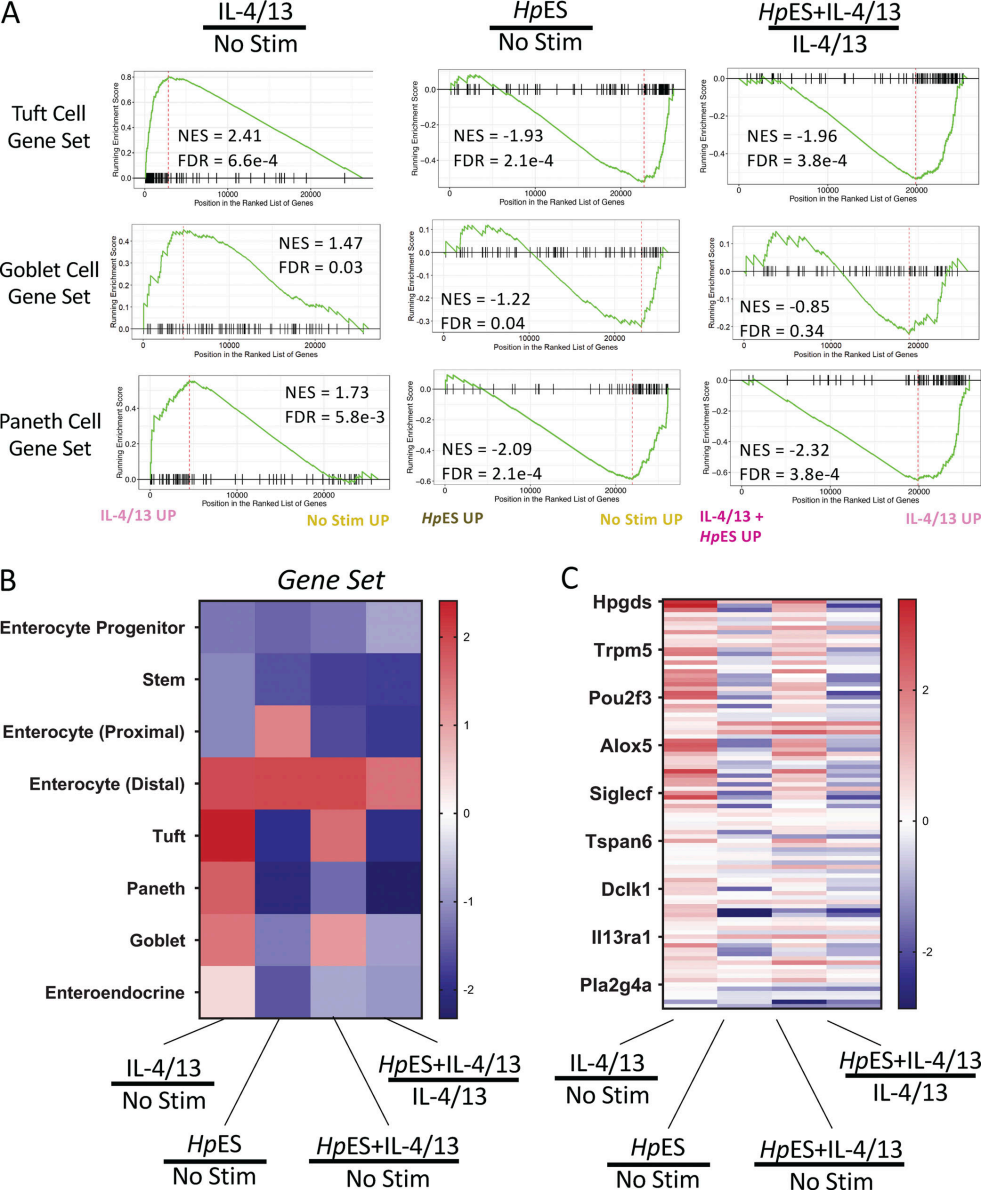
**GSEA of *Hp*ES modulated gene expression. (A)** GSEA of the genes expressed by organoids exposed to IL-4/13, *Hp*ES, or neither, for gene sets of tuft cells, goblet cells, and Paneth cells as described by Haber et al. (2017). Graphs depict the enrichment score (y axis, green line) with positive values where gene sets are induced, and negative values where they are inhibited. Each vertical bar on the x axis represents an individual gene within the gene set for the stated cell type, and its relative ranking against all genes analyzed. NES and false discovery rate (FDR) are indicated on each graph. Gene expression data are pooled from four independent biological replicates, which were analyzed in parallel by RNA-seq. **(B)** Heat map of NES for cell type gene sets from Haber et al. (2017) in organoids treated with combinations of IL-4/13, *Hp*ES, or neither. **(C)** Heat map of tuft cell gene set expression, with key genes indicated on the y axis, showing log_2_ fold change of genes in organoids treated with combinations of IL-4/13, *Hp*ES, or neither. Stim, stimulation.

To search more systematically for potential cell-specific inhibitory effects, we then applied gene set enrichment analysis (GSEA) on the differentially regulated genes. Using gene sets identified from epithelial cell types in single-cell RNA-seq analyses of the intestinal epithelium (Haber et al., 2017), we found that *Hp*ES treatment caused a marked reduction in normalized enrichment scores (NESs) for goblet, Paneth, and tuft cell–associated gene sets, both alone and in the presence of IL-4/13 (Fig. 2, A and B). Reduced scores for each of these gene sets suggest regulation of intestinal cell differentiation by components of *Hp*ES, as all three are secretory cell types (Chiacchiera, 2019).

**Figure 3.**
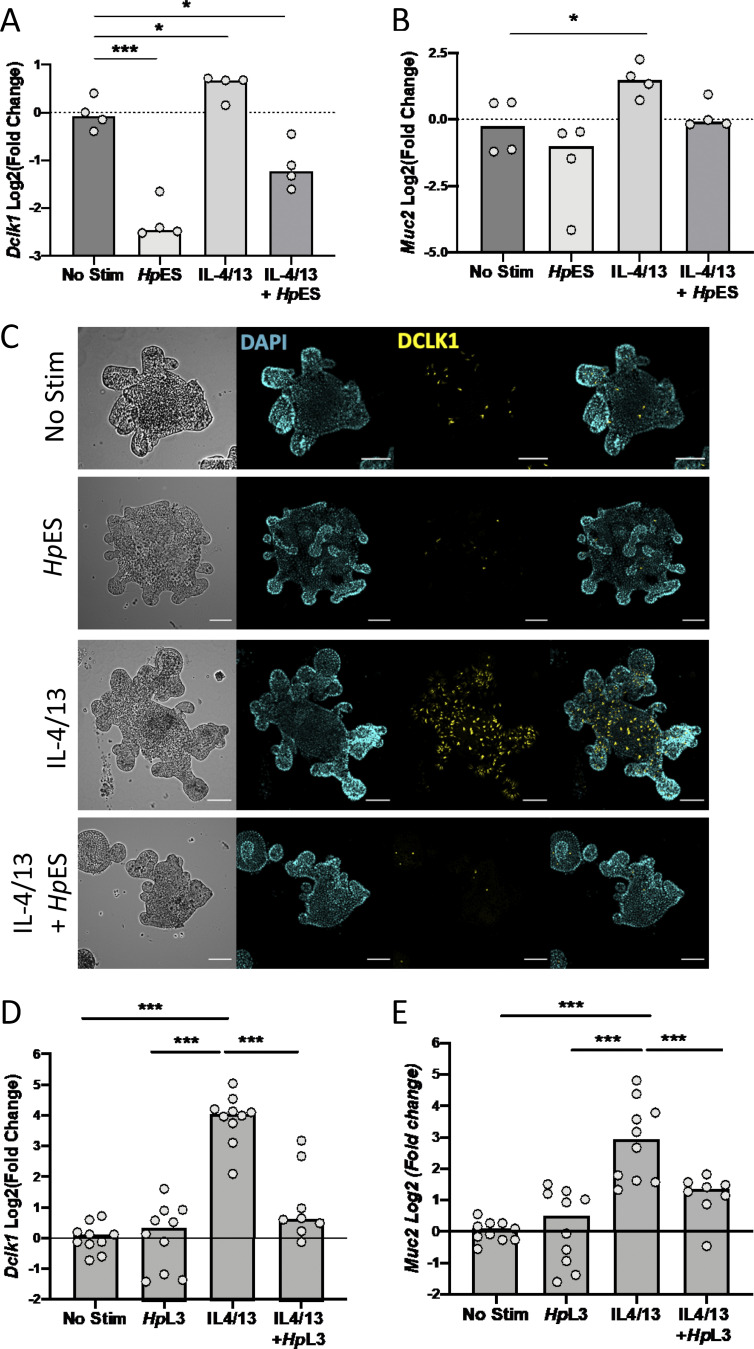
***Hp*ES and *H. polygyrus* larvae repress tuft cell expansion in small intestinal organoids. (A and B)** Expression of canonical tuft cell and goblet cell genes in organoids treated with combinations of IL-4/13, *Hp*ES, or neither. Log_2_ fold change of qRT-PCR values for *Dclk1* and *Muc2* compared with nonstimulated control in four independent biological replicates analyzed by RNA-seq. Statistical analysis was by ordinary one-way ANOVA with Tukey’s multiple comparisons test. *, P < 0.05; ***, P < 0.001. **(C)** Representative images of organoids stained for tuft cells (anti-DCLK1), shown in yellow, in in organoids treated with combinations of IL-4/13, *Hp*ES, or neither. Nuclear staining (DAPI) shown in cyan. Scale bar is 100 µm. **(D and E)** Expression of *Dclk1* and *Muc2* in *H. polygyrus* L3 larvae exposed organoids, Log_2_ fold change of qRT-PCR values shown compared with nonstimulated control. Data are pooled from four experiments each with two to four replicates, total *n* = 8–10 per group. Statistical analysis was by ordinary one-way ANOVA with Tukey’s multiple comparisons test. *, P < 0.05; ***, P < 0.001. Stim, stimulation.

**Figure S3.**
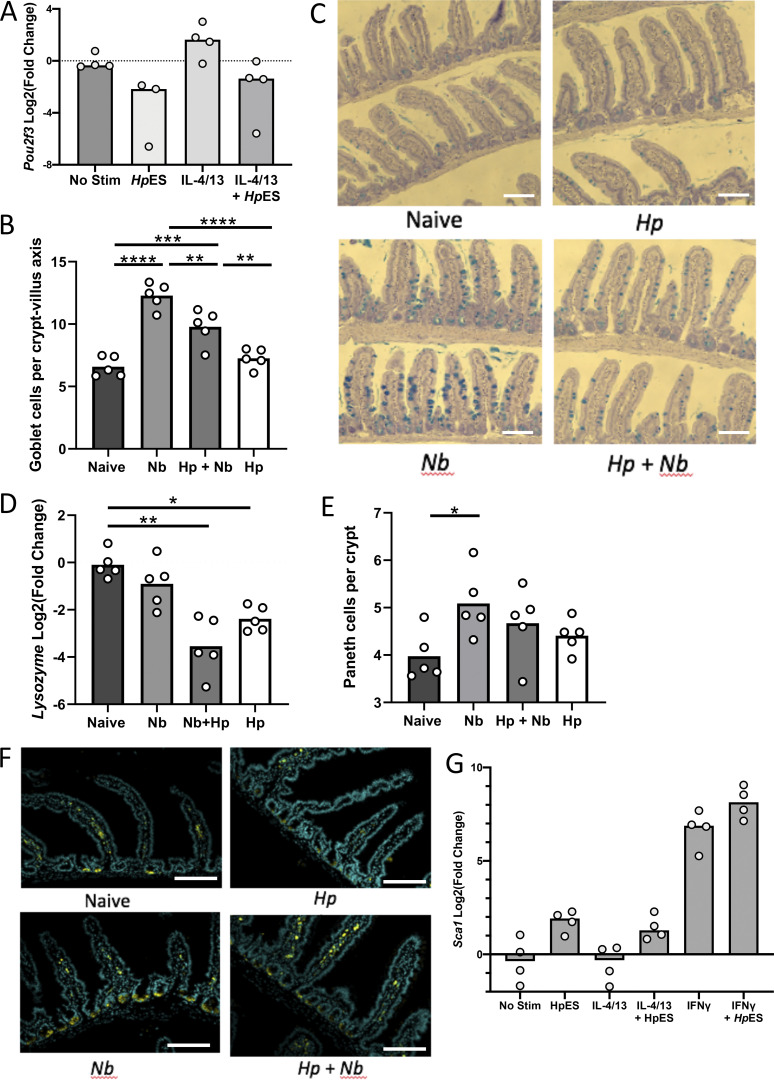
**Cell type–specific inhibition by *Hp*ES and *H. polygyrus.* (A)** *Pou2f3* gene expression in *Hp*ES and IL-4/13 treated organoids. Log_2_ fold change shown compared with nonstimulated control in four separate biological repeats analyzed in parallel by RNA-seq. **(B)** Goblet cell counts in mice singly or coinfected with *H. polygyrus* and/or *N. brasiliensis*, counted villus tip to tip after periodic acid–Schiff staining; 25 counts villus-crypt units were analyzed per mouse. Data are from one experiment with four mice per group, representative of three similar experiments. Kruskal–Wallis test with Dunn’s multiple comparisons test was used; **, P < 0.01; ***, P < 0.001; ****, P < 0.0001. **(C)** Representative images of goblet cell staining using periodic acid–Schiff staining. Scale bar is 100 µm. **(D)** Lysozyme gene expression in intestinal samples. Data are from one experiment with four mice per group, representative of three similar experiments. Ordinary one-way ANOVA with Tukey’s multiple comparisons test was used; *, P < 0.05; **, P < 0.01. **(E)** Paneth cell counts, number of cells per crypt after staining with anti-lysozyme. Data are from one experiment with four mice per group, representative of three similar experiments. Kruskal–Wallis test with Dunn’s multiple comparisons test was used; *, P < 0.05. **(F)** Representative images of Paneth cell staining with anti-lysozyme. Scale bar is 100 µm. **(G)** Expression of *Sca1* from organoid cultures under the indicated conditions. Change shown compared with nonstimulated control in four independent biological replicates analyzed in parallel by RNA-seq. Hp, *H. polygyrus*; Nb, *N. brasiliensis*; Stim, stimulation.

Within the tuft cell gene set, multiple canonical genes such as *Dclk1*, *Pou2f3*, and *Trpm5* showed similar patterns of induction by IL-4/13, but were suppressed by *Hp*ES treatment, even in the presence of type 2 cytokines (Fig. 2 C). Loss of *Dclk1* and *Pou2f3* induction was also confirmed by quantitative RT-PCR (qRT-PCR) analysis of cytokine- and *Hp*ES-treated organoids (Fig. 3 A and Fig. S3 A). Futhermore, *Hp*ES suppressed the induction of *Muc2*, a major gene product of goblet cells (Fig. 3 B). In addition, organoids stained with anti-Dclk1 antibody showed that *Hp*ES completely blocked tuft cell induction by IL-4/13, and even reduced baseline tuft cell differentiation in unstimulated organoids (Fig. 3 C).

*Hp*ES is derived from adult worms collected from the lumen of the intestine. We were interested in whether infective L3 larvae of *H. polygyrus,* which burrow through the epithelium and establish in submucosal tissue, had a similar effect. Adding L3 larvae to the organoids similarly inhibited the induction of *Dclk1* in response to IL-4/13 (Fig. 3 D), suggesting *H. polygyrus* is capable of inhibiting tuft cells at different stages of its life cycle within the host. Furthermore, L3 larvae recapitulated the effect of *Hp*ES in suppressing *Muc2* expression in organoids (Fig. 3 E).

### *H. polygyrus* inhibits tuft cell expansion in vivo

**Figure 4.**
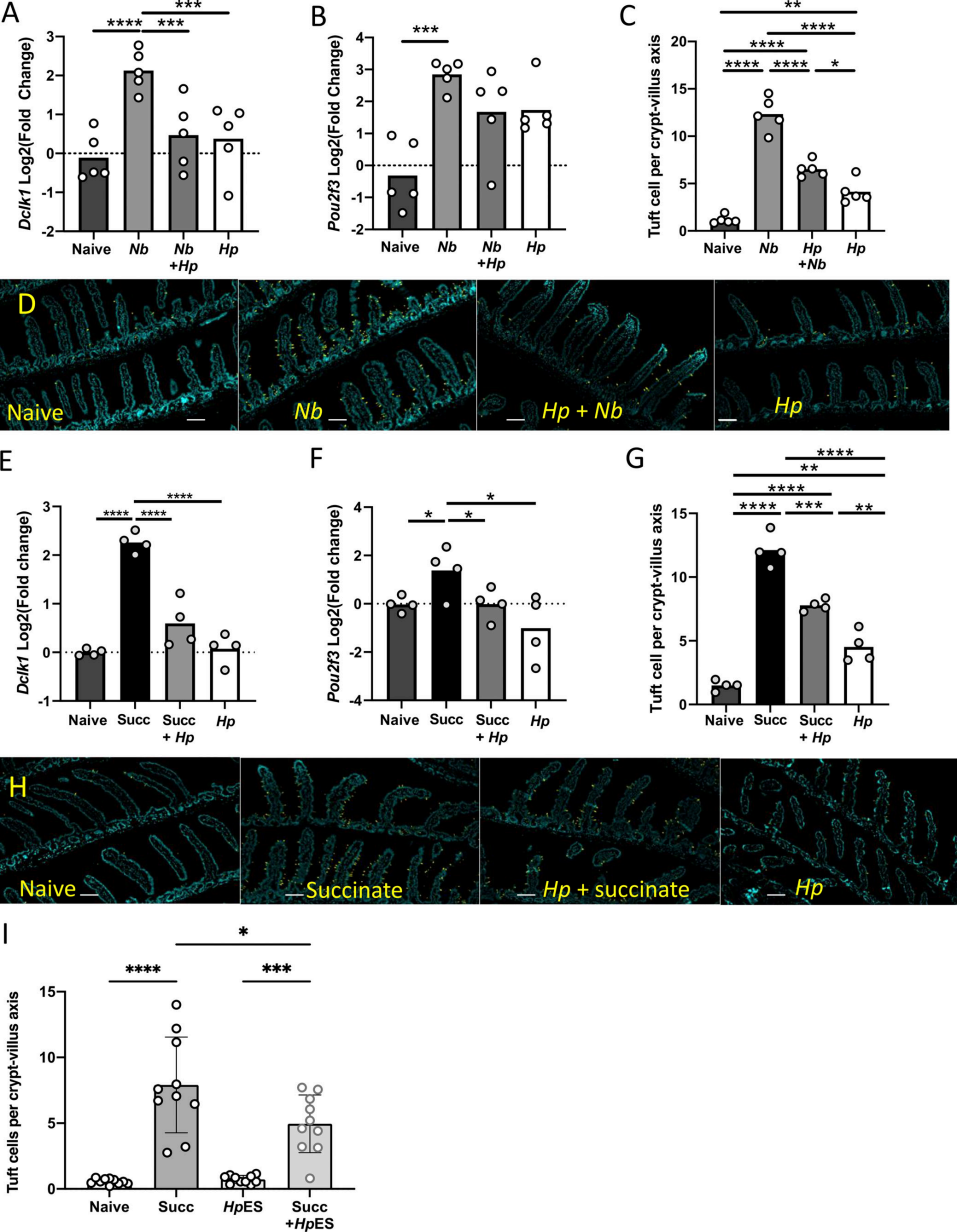
***H. polygyrus* inhibits tuft cell expansion in vivo*.*** Mice were infected with 200 *H. polygyrus* L3 for 28 d before infection with *N. brasiliensis* for 7 d (A–D) or treatment with 100 mM succinate in drinking water for 7 d. Intestinal tissues were taken at day 7 for mRNA isolation and immunohistological analysis. **(A and B)** Expression of canonical tuft cell genes *Dclk1* (A) and *Pou2f3* (B) measured by qRT-PCR in intestinal samples taken from the singly and coinfected mice, presented as log_2_ fold change compared with uninfected controls. Statistical analysis was by ordinary one-way ANOVA with Tukey’s multiple comparisons test. Data are from one of three replicate experiments, each with four or five mice per group. ***, P < 0.001; ****, P < 0.0001. **(C and D)** Tuft cell counts (C) and representative images of tuft cell staining (D) in singly and coinfected mice. Scale bar is 100 µm. Tuft cell counts from ≥20 villus/crypt units were averaged per mouse, and the means for each of five mice per group are presented. Experiments were performed three times with similar results, and data from one representative experiment are shown. Kruskal–Wallis test with Dunn’s multiple comparisons test was used as the discrete data gathered will not be normally (Gaussian) distributed. *, P < 0.05; ****, P < 0.0001. **(E and F)** Expression of canonical tuft cell genes *Dclk1* (E) and *Pou2f3* (F) measured by qRT-PCR in intestinal samples taken from succinate and *H. polygyrus–*infected mice, presented as log_2_ fold change compared with untreated and uninfected controls. Statistical analysis was by ordinary one-way ANOVA with Tukey’s multiple comparisons test. Data are from one of two replicate experiments, each with four mice per group. *, P < 0.05; ****, P < 0.0001. **(G and H)** Tuft cell counts (G) and representative images of tuft cell staining (H) from succinate-treated mice. Scale bar is 100 µm. Tuft cell counts are given as mean for each of four mice per group. Data are from one of two replicate experiments, each with four mice per group. Kruskal–Wallis test with Dunn’s multiple comparisons test was used. **, P < 0.01; ***, P < 0.001; ****, P < 0.0001. **(I)** Tuft cell counts from mice administered with succinate and/or *Hp*ES; 5 µg *Hp*ES was given at days −1, 0, +1, +2, +3 and +4, i.p. Results are pooled from two independent experiments each with five mice per group. Kruskal–Wallis test with Dunn’s multiple comparisons test was used. *, P < 0.05; ***, P < 0.001; ****, P < 0.0001. Hp, *H. polygyrus*; Nb, *N. brasiliensis*; Succ, succinate.

To investigate whether inhibition of tuft cell expansion also occurs in vivo, we performed coinfection experiments with the nematode *N. brasiliensis*, a natural rat parasite that is rapidly expelled by mice through strong tuft cell–dependent type 2 immune responses (Gerbe et al., 2016). We first infected mice with *H. polygyrus*, allowing chronic infection to develop, with adults in the intestinal lumen, over 28 d. We then infected with *N. brasiliensis* for 7 d. Expression of the tuft cell markers *Dclk1* and *Pou2f3* was substantially increased above naive controls in *N. brasiliensis*–infected mice, as expected. However, in *H. polygyrus* infection alone or coinfected mice, increases were muted and were not significantly different from naive controls (Fig. 4, A and B). Dclk1^+^ tuft cell numbers increased sharply in *N. brasiliensis* infections, but significantly less so in *H. polygyrus*– and co-infected mice (Fig. 4, C and D). In a similar fashion, *H. polygyrus* infection also reduced the number of goblet cells induced by *N. brasiliensis* (Fig. S3, B and C), suggesting either that tuft cell inhibition reduces the IL-13–dependent stimulation of goblet cells, or that *Hp*ES constituents more broadly affect differentiation of secretory lineage cells. Expression of lysozyme, a Paneth cell marker, was also reduced by *H. polygyrus* infection, though no reduction in Paneth cell number was seen (Fig. S3, D–F).

We also wanted to investigate whether *H. polygyrus* infection could prevent the induction of tuft cells by the metabolite succinate, which can activate tuft cells through the succinate receptor SUCNR1, inducing a type 2 immune response, including proliferation of tuft cells (Nadjsombati et al., 2018; Schneider et al., 2018). Mice carrying a 28-d *H. polygyrus* infection were treated with 100 mM succinate in drinking water for 7 d. Succinate treatment alone caused increased transcription of the tuft cell markers *Dclk1* (Fig. 4 E) and *Pou2f3* (Fig. 4 F), whereas succinate treatment in *H. polygyrus*–infected mice failed to induce these genes above naive levels. Immunohistochemistry of intestinal tissue samples stained with anti-Dclk1 showed a similar suppression of tuft cell responses in infected mice (Fig. 4, G and H).

We also tested whether *Hp*ES, administered in vivo, could recapitulate the suppressive effects of live infection. Mice receiving 5 µg of *Hp*ES i.p. daily during succinate treatment showed a modest but significant reduction in tuft cell numbers (Fig. 4 I). Together these results show that *H. polygyrus* infection and parasite products can inhibit tuft cell expansion induced both by other helminth species and the metabolite succinate.

### *H. polygyrus* alters differentiation of secretory-lineage cell types

**Figure 5.**
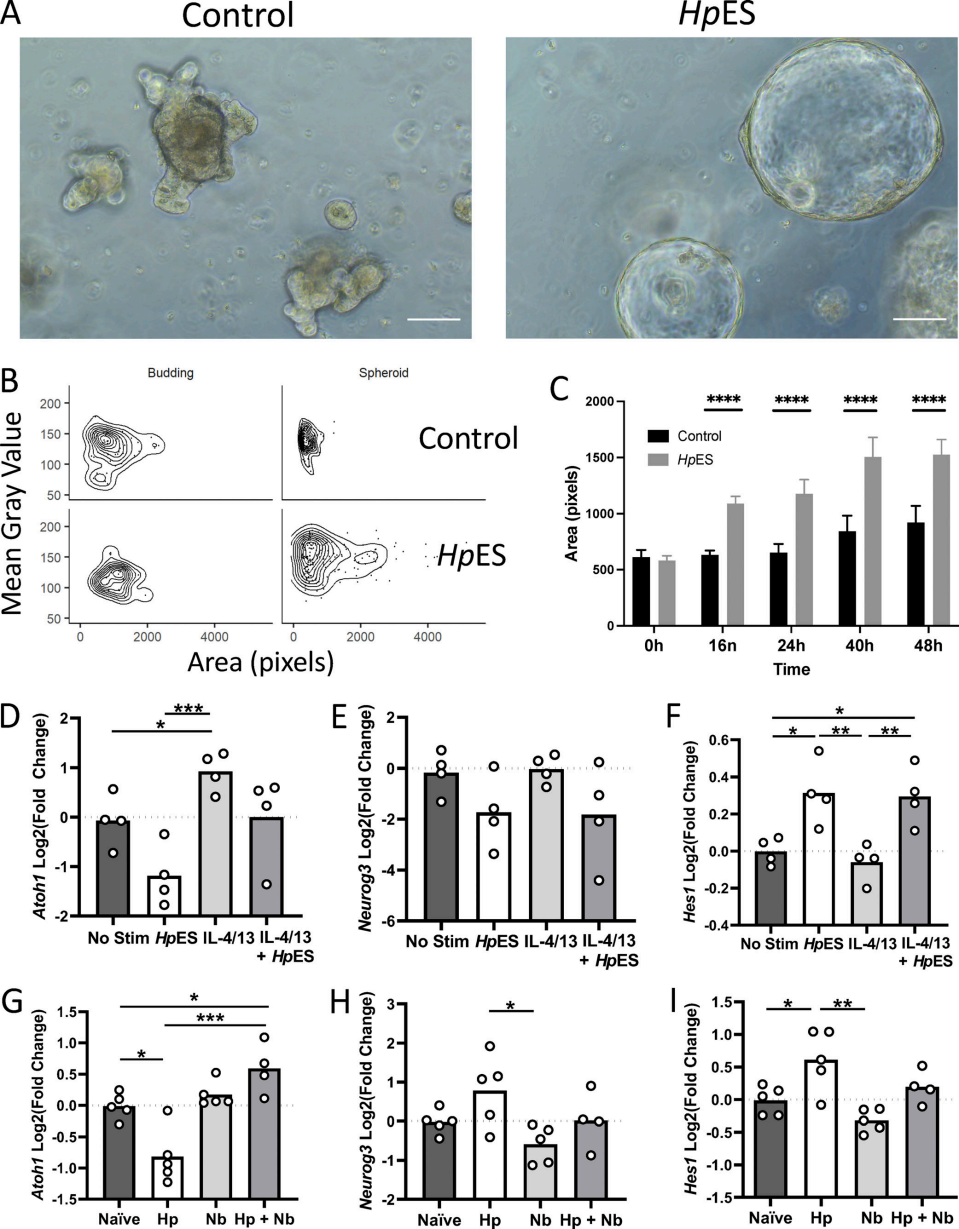
***H. polygyrus* infection alters organoid morphology and the intestinal stem cell niche.** Developmental changes in the intestinal epithelium were investigated by analysis of organoid morphology and key developmental gene expression following *Hp*ES exposure, and expression of the same key genes in intestinal tissue following infection with *H. polygyrus.* **(A)** Images 16 h after culture of control organoids and organoids incubated with *Hp*ES. Scale bar is 100 µm. **(B and C)** Quantification of organoid architecture after addition of *Hp*ES, showing in the distribution of organoids classed as budding or spheroid in the control (top) and *Hp*ES (bottom) treatment conditions (B); and quantification by the area of organoid images over 48 h (C). Unpaired *t* tests were used for statistical analysis; ****, P < 0.0001. **(D–F)** Expression of intestinal development–related genes, *Atoh1, Neurog3*, and *Hes1*, from organoid cultures under the indicated conditions. Change shown compared with nonstimulated control in four independent biological replicates analyzed in parallel by RNA-seq. One-way ANOVA with Tukey’s multiple comparisons test was used; *, P < 0.05; **, P < 0.01; ***, P < 0.001. **(G–I)** qRT-PCR on in vivo intestinal samples for the same intestinal development–related genes. Data shown are from five individual mice in one of three replicate experiments. One-way ANOVA with Tukey’s multiple comparisons test was used; *, P < 0.05; **, P < 0.01; ***, P < 0.001. Hp, *H. polygyrus*;* Nb, N. brasiliensis*; Stim, stimulation.

The inhibition of tuft cell expansion by *H. polygyrus*, as well as inhibition of gene sets for other defense-related cell types by *Hp*ES, suggested a more global effect of this nematode on the intestinal epithelium than compromising individual cell lineages. In this context, we noted marked effects on the morphology of organoids in the presence of *Hp*ES, inducing large spheroid structures with minimal crypt budding when exposed shortly after splitting (Fig. 5 A). Using an automated image analysis pipeline, we were able to categorize organoids as having the immature “spheroid” or mature “budding” morphology (Lindholm et al., 2020 *Preprint*). These results confirmed that *Hp*ES skews organoid morphology toward the enlarged spheroid type (Fig. 5, B and C).

Spheroid organoids have also been observed when grown from stem cells from mice infected with the same parasite, *H. polygyrus* (Nusse et al., 2018); these authors suggested that infection could induce IFN-γ signaling within the epithelium, leading to a fetal-like phenotype of stem cells characterized by *Sca-1* expression that restrains differentiation of secretory cells, while maintaining proliferation (Nusse et al., 2018). Although *Sca-1* expression was highly induced by IFN-γ treatment in our RNA-seq dataset, *Hp*ES treatment had only a minor effect (Fig. S3 G), indicating that *H. polygyrus* modulates intestinal stem cell fate through an IFN-γ– and *Sca-1*–independent pathway.

Due to the changes in morphology induced by *Hp*ES, and the inhibition of diverse secretory lineage cells, we next investigated expression of key developmental genes in the intestinal epithelium. Atoh1, a basic helix-loop-helix (bHLH) transcription factor, is considered to be the master regulator of the secretory cell lineage in the intestinal epithelium, and its expression drives differentiation into the secretory cell types (Yang et al., 2001). Notably, IL-4/13 treatment increases *Atoh1*, but this is ablated in the presence of *Hp*ES (Fig. 5 D), and indeed, *Hp*ES alone decreases *Atoh1* gene expression below levels seen with no stimulation. Genes downstream of *Atoh1*, such as *Neurog3*, which specifies enteroendocrine cells (Jenny et al., 2002), also showed a trend toward suppression by *Hp*ES (Fig. 5 E). *Atoh1* is known to be repressed by another bHLH transcription factor, *Hes1*, which is expressed in proliferating cells and preserves the status of stem cells and transit amplifying cells (Jensen et al., 2000; Yang et al., 2001). While *Hes1* expression is unaffected by IL-4/13, the presence of *Hp*ES treatment markedly increases the transcription of this factor (Fig. 5 F), suggesting a pathway through which *Atoh1* suppression is achieved and secretory cell differentiation inhibited. qRT-PCR from *H. polygyrus*–infected tissues confirmed repression of *Atoh1* expression (Fig. 5 G) and up-regulation of *Hes1* (Fig. 5 I).

Taken together, our results indicate that *H. polygyrus* may redirect the cellular makeup of the intestinal epithelium both by promoting epithelial proliferation (and potentially, repair), as well as reprogramming developmental pathways to favor enterocyte differentiation at the expense of specialized secretory cells that produce mediators detrimental to parasite survival.

It is broadly recognized that helminth parasites modify their environment to optimize their survival, through direct manipulation of host cell signals and responses (Brehm and Koziol, 2017; Yap and Gause, 2018). Previously, attention has mostly focused on modulation of immune system cells and disruption of protective immunity (Gazzinelli-Guimaraes and Nutman, 2018; Maizels et al., 2018; Ryan et al., 2020). However, it is increasingly evident that epithelial cells, specifically stem cells, are affected by Th2 cytokines induced by helminth infection (Biton et al., 2018; Lindholm et al., 2020 *Preprint*). Here, we show that the helminth *H. polygyrus,* and its secreted products, can act directly on the intestinal epithelium that forms its natural niche in vivo. Targeting the epithelium has many advantages for the parasite; it is this tissue that first responds to helminth presence, releasing signals such as thymic stromal lymphopoietin, IL-25, and IL-33. Molecules within *Hp*ES have already been identified that interfere with the IL-33 pathway (Osbourn et al., 2017; Vacca et al., 2020). One of these, HpARI, acts on lung epithelial cells to prevent IL-33 release (Osbourn et al., 2017), and may serve this role in the intestine. In parallel, the suppression of tuft cells will reduce IL-25 in the intestinal milieu, required to trigger ILC2 activation and IL-13 production for type 2 immunity (Gerbe et al., 2016). Indeed, both IL-25–deficient (Neill et al., 2010; Smith et al., 2018) and tuft cell–deficient (Gerbe et al., 2016; Howitt et al., 2016; von Moltke et al., 2016) mice are highly susceptible to intestinal helminth infection.

Tuft cell expansion can be induced by two separate pathways, one defined by the action of succinate on its receptor, SUCNR1, and the second through unknown stimuli emanating from parasitic helminths acting in a SUCNRI-independent fashion (Lei et al., 2018; Nadjsombati et al., 2018; Schneider et al., 2018). The ability of *H. polygyrus* to inhibit tuft cell differentiation in vivo both when exogenous succinate is administered and when a different helminth, *N. brasiliensis*, is introduced, points to a hypothesis that *H. polygyrus* inhibits the development, rather than the function, of tuft cells. Furthermore, replicating the inhibitory effect in vitro in small intestinal organoid cultures confirms that there is a direct helminth–epithelial interaction to dictate cellular differentiation that gives rise across the secretory cell lineages.

Our finding that genes involved in cell fate decisions such as *Atoh1* and *Hes1* are affected by *Hp*ES confirms that this helminth is capable of inducing changes in development. Atoh1 and Hes1 modulate the decision of Lgr5^+^ stem cells to follow the secretory cell type lineage, composed of goblet, Paneth, tuft, and enteroendocrine cells (Chiacchiera, 2019). We found down-regulation of all these gene sets in our RNA-seq data, consistent with the role of other specialized cells in helminth defense (Coakley and Harris, 2020; Sorobetea et al., 2018). Goblet cells are well known for their role in the “weep and sweep” response in helminth defense, producing large quantities of mucins as well as RELM-β, which prevents the worm from locating its food source (Herbert et al., 2009). Paneth cells undergo hyperplasia in response to helminth infection (Kamal et al., 2002), and although primarily associated with anti-microbial immunity, are essential in fostering the Lgr5^+^ stem cell niche (Sato et al., 2011). Similarly, although enteroendocrine cells have no clear role in anti-helminth immunity, they alter in response to helminth infection (Haber et al., 2017; Manocha et al., 2013; Worthington et al., 2018; Worthington et al., 2013), producing serotonin to promote intestinal contractility and the “sweep” part of immunity to helminth infection (Coakley and Harris, 2020; Wang et al., 2018; Zhao et al., 2006). By targeting the whole secretory cell lineage, *H. polygyrus* may coordinately neutralize each of these functions required for effective helminth defense.

Other helminth parasites are known to influence the epithelium of the organ they inhabit, primarily in pro-tumorigenic settings thought to arise as side effects of metabolite release, induction of inflammation, or alterations to commensal bacteria populations (Brindley and Loukas, 2017; Scholte et al., 2018). Interestingly, the human liver fluke *Opisthorchis viverrini*, which can cause cholangiocarcinoma, secretes a granulin-like growth factor, *Ov*-GRN-1, that induces proliferation of host cells (Smout et al., 2009; Smout et al., 2015). Our findings therefore build on these earlier reports that helminth interactions with epithelial tissues are central to the processes of invasion and pathogenesis in parasite infections. We are now endeavoring to identify the *H. polygyrus*–derived factor(s) that mediate the recasting of intestinal epithelial cell fate, to shed greater insight into the host pathways exploited by the parasite, and potentially to discover new molecular tools that can be used to modulate intestinal cell differentiation.

Accompanying the suppression of the secretory cell lineage, we also observed that organoid cultures exposed to *Hp*ES formed a greater proportion of spheroids, strikingly similar to the morphology of organoids produced from stem cells collected from *H. polygyrus*–infected mice (Nusse et al., 2018); in this case, the authors proposed that IFN-γ–induced transcriptional changes, reflecting an absence of Lgr5^+^ stem cells, replaced by Sca1^+^ cells in the infected tissues, led to a fetal reversion of the stem cells. However, our ability to create spheroids in vitro by *Hp*ES exposure argues that host IFN-γ is not required, and that developmental changes are due to the direct effects of secreted factors from the parasite. More broadly, it is interesting to speculate that the spheroid phenotype represents a pro-proliferative tissue repair process that may be essential to recover from parasite migration and epithelial disruption during this infection, raising the possibility that *H. polygyrus* has evolved to mitigate damage and prolong survival of the host in its own interest. Taken together, our findings illustrate the complex relationship between immunity and development at the epithelium, which are manipulated by a sophisticated parasitic helminth to its own advantage and perhaps also that of its host.

## Materials and methods

### Mice and parasites

8–12-wk-old female C57BL/6 mice bred in-house or purchased from Envigo UK housed in individually ventilated cages were used throughout this study. All animal studies were performed under UK Home Office Licence and approved by the University of Glasgow Ethical Review Board.

Infections employed *H. polygyrus* and *N. brasiliensis*, both maintained as previously described (Camberis et al., 2003). *Hp*ES was collected as previously described (Johnston et al., 2015). For succinate experiments, sodium succinate dibasic hexahydrate (Sigma-Aldrich) was dissolved in autoclaved tap water to 100 mM, filter-sterilized, and given to mice as their drinking water. To administer *Hp*ES in vivo, 5 µg in 100 µl PBS was given i.p. daily from day −1 to day +4 relative to addition of succinate to drinking water.

### Organoid culture

Organoids were cultured from crypts isolated from the proximal 10 cm of small intestine (duodenum). Briefly, the duodenum was cut into 2-mm pieces and washed three times with cold PBS before incubation with 2 mM EDTA in PBS for 30 min at 4°C. After removal of EDTA solution, cold PBS was added and crypts isolated from basal membrane by pipetting. This procedure was repeated with more vigorous pipetting to create six fractions. Fractions with enriched crypts were identified using microscopy and pooled through a 70-µm cell strainer (Greiner), then spun at 300 ×*g* for 3 min at 4°C to pellet crypts. Crypts were resuspended and spun at 100 ×*g* for 3 min at 4°C to remove single cells. The pellet was then resuspended in 10 ml basic medium (Advanced DMEM/F12, supplemented with 1% PenStrep, 1% L-glutamine, and 10 mM Hepes, all from Gibco) and crypt numbers counted by microscopy. 500 crypts in 40 µl Matrigel (Corning) were seeded into wells of a 24-well flat-bottomed plate (Corning). After incubation for 10 min at 37°C, 400 µl of complete crypt medium was added (basic medium plus 1× N2 supplement [Life Technologies], 1× B27 supplement [Life Technologies], 50 ng/ml murine epidermal growth factor [Invitrogen], 100 ng/ml murine Noggin [Peprotech], and 500 ng/ml murine R-spondin-1 [R&D]; 3 µM CHIR99021 [Miltenyi] was added only for initial plating). Crypts were then cultured at 37°C in 5% CO_2_, medium was changed every 2–3 d, and organoids were passaged once a week by dissociation and washing in cold basic medium, reseeding at 500 crypts per well in 40 µl Matrigel. Organoids were passaged at least three times before use. Stimulations were as follows: *Hp*ES at 10 µg/ml, IL-4 (Miltenyi) at 400 ng/ml, IL-13 (Peprotech) at 400 ng/ml, and IFN-γ at 1 ng/ml, or 500 *H. polygyrus* L3 stage larvae. Organoids were stimulated for 24 h before collection for RNA-seq.

### RNA extraction

Tissues were taken and stored in either 400 µl TRIzol (Invitrogen; for organoids) or 1 ml RNAlater (Qiagen; for intestine samples) until RNA extraction using the RNeasy mini kit (Qiagen) could be performed. For intestinal samples, tissue was disrupted using a TissueLyser (Qiagen) in 600 µl lysis buffer (RLT, Qiagen) for 2 min at 25 Hz before continuing according to kit instructions. For organoid samples, after spinning at 12,000 ×*g* for 2 min to remove debris, 100 µl chloroform was added to the supernatant before mixing well. After incubation at room temperature for 3 min, samples were centrifuged at 12,000 ×*g* for 15 min at 4°C. The upper aqueous phase was transferred to a new tube and 1.5 volumes of 100% ethanol added before transfer to a RNeasy spin column and purification as per kit instructions. An on-column DNA digestion step was included in RNA purification using a RNase free DNase (Qiagen). RNA concentration was determined using a Nanodrop 2000 (Thermo Fisher Scientific).

### RNA-seq

Library preparation and whole transcriptome profiling was performed by Glasgow Polyomics. PolyA selection library preparation was performed using the TruSeq stranded mRNA kit (Illumina) before single-end sequencing was performed with 30 million reads per sample, 1 x75 nt read length, on the NextSeq 500 platform (Illumina), with results returned as fastq files. Trim Galore! was used to cut adapters (using Cutadapt) and run quality control (using FastQC), with MultiQC used to inspect all samples together. HISAT2 was used to align reads to the mouse genome, followed by featureCounts to aggregate mapped reads. Count data were then analyzed using DESeq2 to identify differential gene expression analysis (Love et al., 2014).

The software degPatterns, within the DEGreport package (Pantano et al., 2020), was used to identify clusters within DEGs, and GO enrichment analysis was performed using enrichGO from clusterProfiler, followed by removal of redundant GO terms using ReViGO (Supek et al., 2011). Heatmaps were created with the aid of the Pheatmap package (Kolde, 2015). UpSet plots were generated using the UpSetR package (Conway et al., 2017). ggplot2 was used for generation of graphs (Wickham, 2016). The R package clusterProfiler was used for GSEA analysis with 10,000 permutations and otherwise default settings.

All RNA-seq data have been submitted to the European Nucleotide Archive with the ArrayExpress accession no. E-MTAB-11118.

### qRT-PCR

Samples used for qRT-PCR were reverse-transcribed using the qScript cDNA synthesis kit (Quanta Bio) according to the kit instructions. cDNA was then diluted 1/10 for use in 12.5 µl qRT-PCR reactions with PerfeCTa SYBR Green Supermix (Quanta Bio) and appropriate primers as previously published (Aronson et al., 2014; Guo et al., 2018; Nusse et al., 2018), detailed in Table 1. Reactions were performed in a 384-well plate (Applied Biosystems) on the QuantStudio 7 Flex real-time PCR system (Applied Biosystems). Analysis was performed using the delta-delta Ct method, with target gene expression normalized against two reference genes. The best reference genes were determined from a selection using the NormFinder plugin for Microsoft Excel (Andersen et al., 2004).

**Table 1.** Primers used for qRT-PCR reactions

Name	Use	Sequence
GAPDH	Reference ER	F 5′-ATG​ACA​TCA​TCA​AGA​AGG​TGG​TG-3′
		R 5′-CAT​ACC​AGG​AAA​TGA​GCT​TG-3′
HPRT	Reference	F 5′-TTT​ACT​GGC​AAC​ATC​AAC​AG-3′
		R 5′-CAG​ATC​CCA​CAT​ACT​CAC​TG-3′
RPS29	Reference	F 5′-ACG​GTC​TGA​TCC​GCA​AAT​AC-3′
		R 5′-CAT​GAT​CGG​TTC​CAC​TTG​GT-3′
Pou2f3	Tuft cell gene	F 5′-AGA​GAA​TCA​ACT​GCC​CCG​TG-3′
		R 5′-GGA​AGG​CAC​GAC​TCT​CTT​CC-3′
DCLK1	Tuft cell gene	F 5′-CAG​CCT​GGA​CGA​GCT​GGT​GG-3′
		R 5′-TGA​CCA​GTT​GGG​GTT​CAC​AT-3′
Muc2	Goblet cell gene	F 5′-CAG​TTT​ATT​CCT​GTG​TGC​CCA​AGG-3′
		R 5′-GGC​TTC​AGA​ATA​ATG​TAC​TGC​TGC-3′
Lysozyme	Paneth cell gene	F 5′-GGA​ATG​GAT​GGC​TAC​CGT​GG-3′
		R 5′-CAT​GCC​ACC​CAT​GCT​CGA​AT-3′
Sca1	Stem cell gene	F 5′-GAT​GGA​CAC​TTC​TCA​CAC​TAC​A-3′
		R 5′-GCA​GGT​AAT​TGA​TGG​GCA​AGA-3′
Lgr5	Stem cell gene	F 5′-CTC​CAA​CCT​CAG​CGT​CTT​C-3′
		R 5′-GTC​AAA​GCA​TTT​CCA​GCA​AGA-3′
Atoh1	Cell fate gene	F 5′-AGC​TTC​CTC​TGG​GGG​TTA​CT-3′
		R 5′-TTC​TGT​GCC​ATC​ATC​GCT​GT-3′
Neurog3	Cell fate gene	F 5′-CTC​AGC​AAA​CAG​CGA​AGA​AG-3′
		R 5′-GGG​AAG​GTG​GGC​AGG​AC-3′
Hes1	Cell fate gene	F 5′-AGT​GTC​ACC​TTC​CAG​TGG​CT-3′
		R 5′-TGG​GCT​AGG​GAC​TTT​ACG​GG-3′

### Organoid immunofluorescent staining

Organoids were seeded out in 4- or 8-well chamber slides (Thermo Fisher Scientific) before use in stimulations and microscopy. Organoids were stimulated for 48 h before use. For immunofluorescence staining, cultured organoids were washed in PBS twice and then fixed in 4% paraformaldehyde for 20 min at room temperature. Organoids were then permeabilized with PBS containing 0.5% Triton X-100 for 10 min at 4°C, followed by rinsing three times with PBS containing 100 mM glycine. Organoids were blocked with IF buffer (PBS containing 0.1% BSA, 0.2% Triton X-100, 0.05% Tween-20, and 10% FCS) for 1 h at room temperature before incubation with 1/1,000 anti-mouse Dclk1 (Abcam) in antibody diluent (Invitrogen) at 4°C overnight. After rinsing three times with IF buffer, slides were incubated with anti-rabbit FITC (Dako) for 1 h at room temperature. Slides were then washed with IF buffer followed by three rinses with PBS before being mounted with Vectashield mounting media containing DAPI (Vector Laboratories). Slides were imaged using a Leica DMi8 inverted microscope and Leica Application Suite (LAS) X software (Leica Microsystems). Resulting image files were analyzed using ImageJ/Fiji (Schindelin et al., 2012).

### Immunohistochemistry

The small intestine was taken and prepared for processing and embedding in paraffin using the Swiss-rolling technique (Bialkowska et al., 2016), which enables many villi to be investigated in the same cut section. Transverse sections were made using a microtome through the gut rolls at a thickness of 5 µm before mounting on glass slides. Sections were deparaffinized by immersing slides in xylene, then hydrated through 100%, 90%, and 70% ethanol successively. Heat-induced epitope retrieval was performed in citrate buffer (Thermo Fisher Scientific), and then sections were blocked using 2.5% normal horse serum blocking solution (Vector Laboratories) for 1 h at room temperature. Slides were then incubated overnight at 4°C with rabbit anti-mouse Dclk1 (Abcam) or rabbit anti-mouse lysozyme (Abcam) at 1:1,000 in 2.5% normal horse serum blocking solution. Polyclonal rabbit IgGs (Abcam) were used as an isotype control. After washing, sections were incubated with swine anti-rabbit-IgGs/FITC (Agilent Dako), washed, and mounted using Vectashield Vibrance antifade mounting medium with DAPI (Vactor Laboratories). Slides were imaged using a Leica DMi8 inverted microscope and Leica Application Suite (LAS) X software (Leica Microsystems). The resulting image files were analyzed using ImageJ/Fiji.

### Periodic acid–Schiff staining

Slides were prepared, deparaffinized, and hydrated as above before undergoing periodic acid–Schiff staining, following the instructions in the kit (Atom Scientific).

### Statistics

All statistical analysis was performed using Prism 8 (Graphpad Software Inc.). Error bars on graphs display the mean and SEM. Ordinary one-way ANOVA with Tukey’s multiple comparisons test was used. In the case of tuft cell count data, a Kruskal–Wallis test with Dunn’s multiple comparisons test was used as the discrete data gathered is not normally (Gaussian) distributed; *, P < 0.05; **, P < 0.01; ***, P < 0.001; and ****, P < 0.0001.

### Online supplemental material

Fig. S1, Fig. S2, and Fig. S3 present additional data on the GO terms most affected by *Hp*ES and cytokines, and the seven clusters of DEGs (Fig. S1); the GO terms most altered within each cluster, and the individual genes most associated with each cluster (Fig. S2); and changes in tuft cell, Paneth cell, and goblet cell differentiation in singly and coinfected mice (Fig. S3).
